# NKG7 Enhances CD8+ T Cell Synapse Efficiency to Limit Inflammation

**DOI:** 10.3389/fimmu.2022.931630

**Published:** 2022-07-06

**Authors:** Emily J. Lelliott, Kelly M. Ramsbottom, Mark R. Dowling, Carolyn Shembrey, Tahereh Noori, Conor J. Kearney, Jessica Michie, Ian A. Parish, Margaret A. Jordan, Alan G. Baxter, Neil D. Young, Amelia J. Brennan, Jane Oliaro

**Affiliations:** ^1^Centre for Cancer Immunotherapy, Cancer Research Division, Peter MacCallum Cancer Centre, Melbourne, VIC, Australia; ^2^Sir Peter MacCallum Department of Oncology, Faculty of Medicine, Density and Health Sciences, The University of Melbourne, Parkville, VIC, Australia; ^3^College of Public Health, Medical & Veterinary Sciences, James Cook University, Townsville, QLD, Australia; ^4^Central Clinical School, Monash University, Prahran, VIC, Australia; ^5^Department of Veterinary Biosciences, Melbourne Veterinary School, Faculty of Veterinary and Agricultural Sciences, The University of Melbourne, Parkville, VIC, Australia

**Keywords:** NKG7, cytotoxic T lymphocytes, immune synapse, tumor necrosis factor, immunotherapy, inflammation

## Abstract

Cytotoxic lymphocytes are essential for anti-tumor immunity, and for effective responses to cancer immunotherapy. Natural killer cell granule protein 7 (NKG7) is expressed at high levels in cytotoxic lymphocytes infiltrating tumors from patients treated with immunotherapy, but until recently, the role of this protein in cytotoxic lymphocyte function was largely unknown. Unexpectedly, we found that highly CD8+ T cell-immunogenic murine colon carcinoma (MC38-OVA) tumors grew at an equal rate in *Nkg7^+/+^* and *Nkg7^-/-^* littermate mice, suggesting NKG7 may not be necessary for effective CD8+ T cell anti-tumor activity. Mechanistically, we found that deletion of NKG7 reduces the ability of CD8+ T cells to degranulate and kill target cells *in vitro*. However, as a result of inefficient cytotoxic activity, NKG7 deficient T cells form a prolonged immune synapse with tumor cells, resulting in increased secretion of inflammatory cytokines, including tumor necrosis factor alpha (TNF). By deleting the TNF receptor, TNFR1, from MC38-OVA tumors, we demonstrate that this hyper-secretion of TNF compensates for reduced synapse-mediated cytotoxic activity against MC38-OVA tumors *in vivo*, *via* increased TNF-mediated tumor cell death. Taken together, our results demonstrate that NKG7 enhances CD8+ T cell immune synapse efficiency, which may serve as a mechanism to accelerate direct cytotoxicity and limit potentially harmful inflammatory responses.

## Introduction

Tumor infiltrating cytotoxic lymphocytes, such as CD8+ T cells and natural killer (NK) cells, are essential for effective anti-tumor immunity. Upon recognition of cognate antigen, cytotoxic CD8+ T cells form an immune synapse with target tumor cells which triggers their effector activity (1). This is characterized by directed polarization of cytotoxic lytic granules to the interface of the two cells and the release of perforin and granzymes, leading to apoptosis of the tumor cells (2). At the same time, the T cells secrete pro-inflammatory cytokines, such as interferon-gamma (IFN-γ) and tumor necrosis factor alpha (TNF) (3), which can have direct cytotoxic activity on tumor cells, as well as promoting further anti-tumor immune activity (4, 5).

The number of tumor infiltrating cytotoxic CD8+ T cells positively correlates with disease outcome in a number of cancer types (6), including breast (7, 8) and colon (9). CD8+ T cells also contribute to the efficacy of many cancer therapies (10), so approaches that enhance their activity within tumors through antagonism of inhibitory checkpoint receptors, such as PD-1 or CTLA-4, are showing great success in the clinic and revolutionizing outcomes for patients with otherwise incurable metastatic disease (11, 12). However, a limitation to this approach is that the number and functionality of the T cells can vary significantly within tumors, leading to wide variation in the proportion of patients who respond positively to this therapy.

We, and others, have recently applied advanced single cell RNA sequencing technology to interrogate tumor infiltrating lymphocytes, which has enabled the molecular signatures associated with the different T cell subsets found within both mouse (13–15) and human tumors (16) to be intricately defined. Similar studies have revealed that particular CD8+ T cell subsets within tumors are essential for durable and effective responses to cancer immunotherapies such as checkpoint blockade (17, 18). A number of these studies have highlighted that expression of the gene encoding natural killer granule associated protein 7 (*Nkg7*) in cytotoxic lymphocytes is associated with anti-tumor immunity (17, 19, 20), and reignited interest in this protein as a therapeutic target for improving cancer immunotherapy (20, 21).

NKG7 was first described as an intrinsic membrane protein associated with the cytotoxic granules of natural killer (NK) cells almost two decades ago (22, 23). These early studies reported that NKG7 translocates to the plasma membrane upon target cell induced degranulation, suggesting that it may facilitate the cytotoxic activity of both T and NK cells. However, until recently, little was known about the intrinsic role of this protein in cytotoxic lymphocytes, despite it being highly expressed in cells with cytotoxic function, including natural killer cells, cytotoxic CD8+ T cells and cytotoxic CD4+ T cells associated with viral infections (24). More recently, a role for NKG7 in regulating anti-tumor immunity has been reported (20, 21, 25). These studies have collectively demonstrated that loss of NKG7 impairs both NK and CD8+ T cell-mediated anti-tumor control *in vivo* and may be important for effective CD8+ T cell responses following immune checkpoint blockade in both mouse and human models (20, 21).

In this study, we add to the growing literature supporting a role for NKG7 in modulating CD8+ T cell cytotoxic activity against tumor cells. We show that NKG7 deficient CD8+ T cells (from NKG7 deficient mice or following CRISPR/Cas9 editing) fail to effectively kill target cells *in vitro*, resulting in prolonged immune synapse formation and hypersecretion of cytokines, including TNF. Importantly, *in vivo*, the hypersecretion of TNF by NKG7 deficient CD8+ T cells compensates for reduced perforin-mediated control of MC38-OVA tumors through enhanced TNF-mediated tumor cell death. Our data highlights the complex role of NKG7 in both direct tumor cell lysis and inflammatory responses underscoring CD8+ T cell anti-tumor immunity.

## Materials and Methods

### Mice and Cells

All animal studies were performed in accordance with the NHMRC Australian Code for the Care and Use of Animals for Scientific Purposes 8th edition (2013) and with approval from the Peter MacCallum Cancer Centre Animal Experimentation Ethics Committee. C57BL/6J *Nkg7^-/-^* and C57BL/6J wildtype mice were kindly supplied by Professor Alan Baxter (James Cook University, QLD, Australia) intercrossed and bred in the Peter MacCallum Cancer Centre Animal Core Facility under specific pathogen-free conditions to generate C57BL/6J *Nkg7^+/+^* and *Nkg7^-/-^* littermates for these studies. Genotypes were determined by PCR using the following primers to identify the 286bp WT allele:

Reg-Nkg7 WT F (5’- AGACTCAAGTAGCAGGTAAAGGGGC-3’)

Reg-Nkg7 WT R (5’- CAGGATTCACCAGTCTAGGTGTCCC-3’)

and the following primers to identify the 465bp Nkg7 knockout allele:

Reg-Neo F (5’- GCAGCCTCTGTTCCACATACACTTCA-3’)

Reg-Nkg7 R (5’-TTGAGGTAGGGTCTCACTACGTTGC-3’)

MC38-OVA and P815 tumor cell lines were cultured in DMEM containing 10% FBS and cultured at 37°C in 10% CO_2_. All cell lines were confirmed negative for mycoplasma by PCR. MC38-OVA-*Tnfrsf1a^-/-^* cells were generated by CRISPR/Cas9 editing as previously described (26). Primary T cells were cultured in RPMI supplemented with 10% FBS, 1% GlutaMAX, 1 mM Sodium Pyruvate, 1% MEM Non-Essential Amino Acids, 0.1% 2-mercaptoethanol and 100 IU/mL recombinant human IL-2 (NIH), and cultured at 37°C in 5% CO_2_.

### Antibodies and Reagents

The antibody clones used for flow cytometry were CD8 (BioLegend, 53-6.7), CD62L (eBioscience, MEL-14), CD44 (eBioscience, IM7) CD107a/LAMP1 (BD Biosciences, H4A3) and NKG7 (Cell Signaling Technology, E6S2A); for microscopy were EEA1 (Cell Signaling Technology, E9Q6G), Rab27 (Cell Signaling Technology, E907E), CD107a/LAMP1 (BioLegend, H4A3) Granzyme B (BD Biosciences, GB11) and rabbit and mouse anti-tubulin (Rockland). Secondary antibodies conjugated to Alexa Fluorophores, ProLong™ Gold Antifade Mountant with DAPI, CellTrace™ Violet and CFSE dyes were purchased from Molecular Probes (Thermo Fisher). Calcein-AM and LysoTracker™ were purchased from Invitrogen (Thermo Fisher).

### Functional T Cell Assays

Naïve CD8+ T cells were isolated from the spleens of *Nkg7^+/+^* and *Nkg7^-/-^* littermate mice using EasySep™ Mouse Naïve CD8+ T cell Isolation Kit (StemCell Technologies, 19858) and activated on tissue culture plates that had been coated with anti-CD3 (1 ug/mL) and anti-CD28 (2 ug/mL) antibodies (BD Biosciences) in PBS overnight at 4°C. The cytotoxic activity of activated T cells was measured by a standard chromium release assay using chromium-labeled tumor cells. The percentage-specific killing was determined using the formula: (Sample ^51^Cr release – Spontaneous ^51^Cr release)/(Total ^51^Cr release – Spontaneous ^51^Cr release) x 100 and represented as a Michaelis–Menten kinetic trend. To generate relative killing bar graphs, relative killing at the E:T ratio that results in 50% maximal killing of the least cytotoxic condition was compared, using Michaelis–Menten trends, as done previously (27). For the degranulation assay, T cells were cultured with targets at an E:T ratio of 4:1, with Golgi Plug and anti-CD107a (BD Biosciences). After 4 hours, cells were washed, then analyzed by flow cytometry. For the conjugation assay, activated T cells were labeled with CellTrace™ Violet and incubated with CellTrace™ CFSE-labeled tumor cells at 37°C. The cells were vortexed to separate any non-antigen-specific conjugates, and the cells fixed in 4% paraformaldehyde and analyzed by flow cytometry for the presence of double-positive conjugates. Cytokines were detected using a mouse inflammation CBA kit (BD Biosciences, 552364) as per manufacturer’s instructions and analyzed on a FACS Symphony (BD Biosciences), or by a mouse inflammation antibody membrane array (40 target cytokines) (Abcam, ab133999) as per the manufacturer’s instructions. All assays were analyzed using triplicate determinations.

### RNA-Sequencing and Analyses

Activated T cells were co-cultured with tumor cells for 4 hours before being sorted and lysed for total RNA extraction. Lysis was achieved using TRIzol reagent (Thermo Fisher Scientific) and total RNA was extracted using a Directzol RNA miniprep kit (Zymo Research) as per the manufacturer’s instructions. The QuantSeq mRNA Library Kit (Lexogen) was used to prepare libraries. Single-end 75 base pair RNA sequencing on mRNA libraries was performed in-house at the Peter MacCallum Cancer Centre Molecular Genomics Core on a NextSeq 500 (Illumina). Demultiplexing of reads was performed using CASAVA (v1.8) and Cutadapt (v1.7) was used to trim polyA-derived sequences and biased reads resulting from random hexamer priming. HISAT2 (v2.1) was used to align the resulting reads to the mouse reference genome, GRCm38/mm10. Read counting was performed using featureCounts from the Subread package (v1.5). Differential gene expression was performed using Voom-LIMMA and gene set enrichment analysis (GSEA) was performed using GSEA2 (v3) for identification of enriched signatures obtained from the MSigDB Hallmark datasets (28). All sequencing data has been deposited in a public, community-supported repository under BioSample (SAMN28461275- SAMN28461279) and Bioproject IDs (PRJNA838721).

For bulk RNA sequencing expression analyses from publicly available datasets (GSE107011 (29); GSE60424 (30); GSE22886 (31)) raw counts files were downloaded from Gene Expression Omnibus (GEO) using the NCBI portal (http://www.ncbi.nlm.nih.gov/geo/). The filterByExpr function from the edgeR package (v 3.28.1) was used to filter lowly expressed genes and calculated count- or transcript-per-million (CPM/TPM) values. All computational analyses were performed using R (version 3.6.1). For data wrangling and visualization, base R functions were used alongside several core packages from the tidyverse (v 1.3.0) R package. tidyr (v 1.1.2) and dplyr (v 1.0.2) were used for reading and manipulating the data, as well as ggplot2 (v 3.2.1) for visualization. For single cell RNA sequencing expression analysis, processed counts from GSE127465 (32) were interrogated and visualized using the Single Cell Portal from the Broad Institute (https://singlecell.broadinstitute.org/single_cell).

Cellular Indexing of Transcriptomes and Epitopes by sequencing (CITE-Seq) was performed on tumour infiltrating lymphocytes from MC38-OVA bearing mice using cell hashing and demultiplexing as previously described (14, 33, 34). Quality control was performed by removing cell barcodes that were outliers by a high mitochondrial gene percentage (>7%), number of detected features in the RNA library (<200 or >3500), number of RNA counts (>20000) or number of antibody-derived tags (ADT) counts (>5000). Normalization, variable feature selection, scaling, principal component analysis, clustering, and dimensionality reduction using Uniform Manifold Approximation and Projection (UMAP) (35) were performed using standard workflows from the *Seurat* package in *R*. Effector and memory gene signature scores were generated using published gene sets (36) and the *AddModuleScore* function in *Seurat*. Clusters were annotated using a combination of ADTs, key gene expression and gene signature scores

### Microscopy

Fixed confocal microscopy: Activated CD8+ T cells from the spleens of *Nkg7^+/+^* and *Nkg7^-/-^* littermate mice were overlaid into eight-well chamber slides (Nalge Nunc) in pre-warmed, serum-free media RPMI-1640, and allowed to settle to the bottom of the well by incubating them at 37°C for 15mins. Media was then gently removed, and cells were fixed with PHEM-buffer [60 mM PIPES, 25 mM HEPES, 10 mM EGTA, 2 mM MgCl_2_, 4% paraformaldehyde) or Bouin’s solution [5% acetic acid, 9% formaldehyde, 0.9% picric acid], permeabilized with 40 uM β-escin (Sigma)/PBS or 0.1% Triton-X/PBS and then labeled with primary antibodies. This was followed by detection with Alexa Fluor–conjugated secondary antibodies and slides were mounted in ProLong™ Gold Antifade containing DAPI as previously described (37). For immune synapse analyses, tumor cells were labelled with CellTrace™ Violet and allowed settle to the bottom of eight-well chamber slides as described above. Activated CD8+ T cells from the spleens of *Nkg7^+/+^* and *Nkg7^-/-^* littermate mice were overlaid for 45 mins and non-adherent cells washed off. Cell conjugates were fixed and permeabilized and stained as above. T cells selected for protein scoring had a single contact site with one tumor cell and polarized microtubule organizing center (MTOC). Slides were examined using a Nikon C2 Confocal Microscope equipped with 405nm/488nm/561nm/640nm laser diodes or Zeiss Elyra PS.1 microscope. All images were processed using Fiji-ImageJ, and Volocity 3D Image Analysis software was used to calculate protein co-localization.

Time-lapse microscopy: Activated CD8+ T cells from the spleens of *Nkg7^+/+^* and *Nkg7^-/-^* littermate mice were labeled with LysoTracker™, pelleted and resuspended in pre-warmed media for 10 mins. Labeled T cells were then added to CellTrace™ Violet-labelled adherent tumor target cells in media containing 100 uM propidium iodide (PI) and imaged as previously described (5, 38). Briefly, chamber slides were mounted on a heated stage within a temperature-controlled chamber maintained at 37°C, and constant CO_2_ concentration of 5% was infused using a gas incubation system with active gas mixer (“The Brick”; Ibidi). Optical sections were acquired through sequential scans or brightfield/DIC on a TCS SP5 confocal microscope (Leica Microsystems) using a 40 (NA 0.85) air objective and Leica LAS AF software. Image analysis was performed using Meta-Morph Imaging Series 7 software (Universal Imaging).

### *In Vivo* Mouse Experiments

C57BL/6J *Nkg7^+/+^* and *Nkg7^-/-^* mice were injected subcutaneously with 1x10^6^ MC38-OVA or MC38-OVA-*Tnfrsf1a*^-/-^ tumor cells. Tumor growth was monitored approximately every second day using a caliper square to determine the product of 2 perpendicular tumor diameters. CD8 depletion antibodies (YTS 169.4) were administered at 200 ug/mouse on days -1, 0 and 7 and weekly ongoing, with day 0 being the day of tumor inoculation. Mice were culled when the tumor size reached the ethical limit (180 mm^2^).

### Statistical Analyses

One-way ANOVA with Tukey multiple comparisons tests, log-rank (Mantel–Cox) test, 2-way ANOVA Sidak’s multiple comparisons and Welch’s t test, Mann-Whitney test and unpaired t tests were performed using GraphPad PRISM. All experiments were performed in at least three biological replicates, unless otherwise specified, and error bars show SEM. Significance was determined as, *P < 0.05; **P < 0.01; ***P < 0.001; ****P < 0.0001.

## Results

### NKG7 Is Highly Expressed in CD8+ Effector T Cells, but Is Not Required for CD8+ T cell-Mediated Tumor Control *In Vivo*

**Figure 1:**
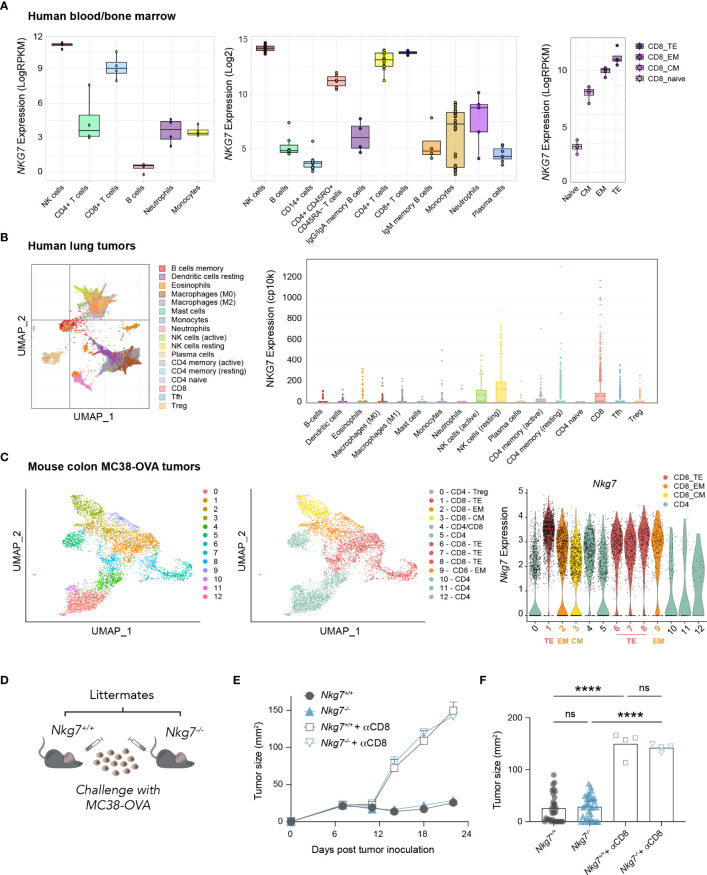
NKG7 is highly expressed in tumor-infiltrating CD8+ effector T cells but is not required for CD8+ T cell-mediated control of MC38-OVA tumors *in vivo*. **(A–C)** Analysis of bulk and single-cell RNA-seq and CITE-seq datasets available through the GEO. TE – Terminal Effector; EM – Effector Memory; CM – Central Memory **(A)** Bulk RNA-seq showing *NKG7* expression on sorted cells from healthy human donor whole blood (left panel; GSE60424 (30), PBMCs or bone marrow (middle panel; GSE22886 (31), and PBMCs (right panel; GSE107011 (29). **(B)** Single cell RNA-seq showing NKG7 expression across different tumor-infiltrating immune subsets in human lung carcinoma (GSE127465 (32). **(C)** Single cell CITE-seq showing *Nkg7* expression in tumor infiltrating T cells from MC38-OVA tumors harvested 10 days post subcutaneous tumor inoculation in C57BL/6 mice (GSE182664 (14). **(D)** Schematic of *in vivo* tumor growth study. MC38-OVA cells were implanted subcutaneously in *Nkg7^+/+^* or *Nkg7^-/-^* littermates with or without CD8 depletion antibodies administered starting the day prior to tumor inoculation. **(E)** Tumor growth, error bars show SEM. **(F)** Tumor size on day 22 post tumor inoculation, data is pooled from 4 independent experiments, One-way ANOVA (n = 4-31). ns – not significant, **** P <0.0001.

Given recent evidence highlighting a role for NKG7 in anti-tumor immunity (20, 21, 25) we examined the expression of *NKG7*/*Nkg7* amongst immune cell subtypes in human and mouse samples using available RNA-sequencing datasets. These data confirmed earlier reports that *NKG7*/*Nkg7* is expressed predominantly in cytotoxic lymphocytes, including natural killer (NK) cells and CD8+ T cells, suggesting a role for this protein in cell-mediated cytotoxicity (**Figures 1A–C**, **Supplementary Figure 1**). Notably, within the CD8+ T cell population in both mouse tumors and human peripheral blood, *Nkg7*/*NKG7* expression was highest in effector subsets and lower in central memory and naïve CD8+ T cells, signifying a potential contribution of NKG7 in CD8+ T cell cytotoxic effector function (**Figures 1A–C**).

To test this, we challenged cohorts of C57BL/6J *Nkg7^+/+^* and *Nkg7^-/-^* littermate mice with the mouse colon carcinoma tumor line, MC38-OVA; a model in which the growth of tumors is acutely controlled by endogenous OVA-reactive CD8+ T cells in the 3 weeks following tumor inoculation (14). Unexpectedly, we found that tumors grew at an equivalent rate in *Nkg7^+/+^* and *Nkg7^-/-^* mice across 4 independent experiments (**Figures 1D–F**). Tumor control in both *Nkg7^+/+^* and *Nkg7^-/-^* mice was confirmed to be CD8+ T cell-mediated, as *in vivo* administration of CD8 depleting antibodies led to rapid tumor outgrowth in all mice (**Figures 1E, F****).** In contrast to findings of a recent study, in which tumor growth in *Nkg7^-/-^* mice was compared to wild-type C57BL/6J mice (21), our results, generated using littermate controls, suggested that NKG7 is not required for effective anti-tumor CD8+ T cell immunity against MC38-OVA tumors *in vivo*.

### NKG7-Deficient CD8+ T Cells Undergo Normal Activation but Exhibit Reduced Cytotoxicity

**Figure 2:**
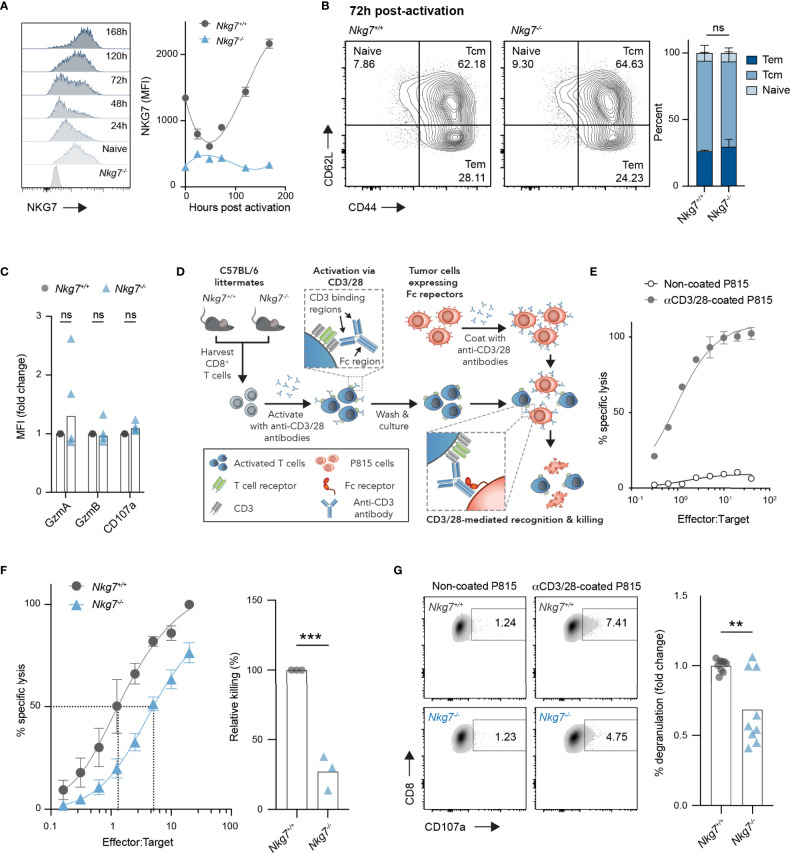
NKG7-deficient CD8+ T cells undergo normal activation but exhibit reduced cytotoxicity. **(A)** NKG7 expression in CD8+ T cells isolated from the spleens of C57BL/6J *Nkg7^+/+^* or *Nkg7^-/-^* littermates, measured by flow cytometry before and at indicated timepoints post-activation with anti-CD3/CD28 antibodies. **(B)** CD8+ T cells from **(A)** evaluated by flow cytometry 72 hours post activation and classified as naïve (CD44^-^CD62L^+^), central memory (Tcm; CD44^+^CD62L^+^) or effector memory (Tem; CD44^+^CD62L^-^), 2way ANOVA Sidak’s multiple comparisons test, n = 3. **(C)** Intracellular protein expression measured by flow cytometry 72 hours post activation, 2way ANOVA Sidak’s multiple comparisons test, n = 4. **(D)** Schematic of *in vitro* cytotoxicity assay. CD8+ T cells were activated as in **(A)** for 72 hours and co-cultured with anti-CD3/28 antibody-coated P815 tumor cells, which express high levels of Fc receptors that bind the Fc region of anti-CD3/28 antibodies. **(E)** Specific lysis of chromium (^51^Cr)-labelled P815 tumor cells (targets) by activated T cells (effectors) in a 4-hour co-culture as measured by chromium release at increasing effector to target ratios. **(F)** Specific lysis of anti-CD3/28-coated P815 cells (targets) by 72 hour activated *Nkg7^+/+^* or *Nkg7^-/-^* CD8+ T cells (effectors) in a 4-hour co-culture. Relative killing calculated as the relative efficiency of T cells to achieve 50% specific lysis of target cells, unpaired t test, n = 3. **(G)** Degranulation of 72 hour activated *Nkg7^+/+^* or *Nkg7^-/-^* CD8+ T cells co-cultured with P815 target cells for 4 hours, measured by CD8+ T cell surface exposure of CD107a during the co-culture, detected by flow cytometry, unpaired t test, n = 9. All error bars show +/- SEM. ns – not significant, ** P < 0.001, *** P < 0.001.

Given the unexpected finding above, we next investigated the intrinsic role of NKG7 in the phenotype and function of CD8+ T cells. We first evaluated expression of NKG7 protein in CD8+ T cells over time following activation with anti-CD3/28 antibodies *in vitro*. Following a small decrease in expression in the first 48 hours post-activation, NKG7 protein levels increased sharply at 72 hours and continued to increase over time in culture (**Figure 2A**). Interestingly, despite such dynamic changes in NKG7 protein expression following activation, the absence of NKG7 in CD8+ T cells from *Nkg7^-/-^* mice had no effect on differentiation into effector memory (CD62L+CD44+) and central memory (CD62L+CD44+) subsets (**Figure 2B**). Likewise, as previously reported (25), we found no significant differences in expression of the effector molecules, Granzyme A and Granzyme B, or the degranulation marker, CD107a, between activated *Nkg7^+/+^* and *Nkg7^-/-^* CD8+ T cells (**Figure 2C**).

To test the cytotoxic activity of CD8+ T cells from *Nkg7^+/+^* and *Nkg7^-/-^* littermate mice in the absence of antigen specificity, we optimized a surrogate method using the Fc receptor-expressing cell line, P815, as targets for T cell-mediated cytotoxicity. To this end, P815 cells were coated with anti-CD3/28 antibodies to promote the formation of an immune synapse with CD8+ T cells *via* direct ligation of CD3, which resulted in CD8+ T cell specific lysis of P815 target cells (**Figures 2D, E****)**. Using this model system, we found that *Nkg7^-/-^* CD8+ T cells were capable of lysing tumor cell targets, but at a significantly reduced efficiency compared to *Nkg7^+/+^* T cells (**Figure 2F**). As previously reported (20, 25), this reduction in cytotoxic activity was associated with reduced degranulation (**Figure 2G****).** Together, these data demonstrate that NKG7 does not influence T cell differentiation, but may have a direct function in enhancing the efficiency of CD8+ T cell degranulation and cytotoxicity.

### NKG7 Is Localized to Late Endosomes and Polarizes to the Immune Synapse Upon Target Recognition

**Figure 3:**
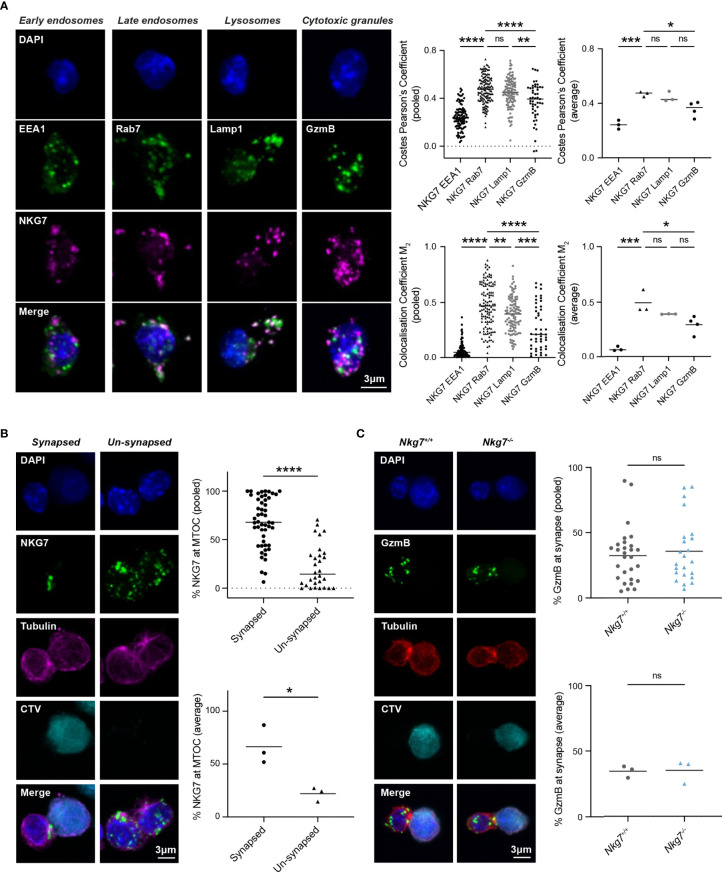
NKG7 protein is localized to late endosomes and polarizes to the immune synapse upon target recognition. **(A)** Confocal immunofluorescence microscopy of *Nkg7^+/+^* CD8+ T cells showing co-localization of NKG7 (magenta) with Lamp-1 and Rab-7 (green; middle panels), but not EEA1 or GzmB (green; far left and right panels). Both Costes Pearson’s Correlation Coefficient and Manders Correlation Coefficient, were used to quantify co-localization (left); biological replicates (right). Comparisons made by one-way ANOVA, each dot represents an individual cell analyzed (n = 47-133) pooled from 3 independent experiments (average shown on right) **(B)** The percentage of NKG7 (green) present at the MTOC (magenta) for synapsed and un-synapsed *Nkg7^+/+^* CD8+ T cells, with synapse determined by polarisation of the MTOC to the interface of the two cells. Anti-CD3/28-coated P815 target cells were labelled with cell trace violet (CTV) and co-cultured with *Nkg7^+/+^* CD8+ T cells for 45 mins. Comparisons made by unpaired t test. Each dot represents an individual cell conjugate analyzed (n = 30-51) pooled from 3 independent experiments (average shown below) **(C)** The percentage of granzyme B (GzmB; green) present at the immune synapse (MTOC; red) in *Nkg7^+/+^* and *Nkg7^-/-^* CD8+ T cells, during co-culture with target cells as described in **(B)**, unpaired t test. Each dot represents an individual cell conjugate analyzed (n = 23-28) pooled from 3 independent experiments (average shown below) * P < 0.05, ** P < 0.01, *** P < 0.001, **** P < 0.0001. NS, not significant.

The reduced degranulation and cytotoxic efficiency of *Nkg7^-/-^* CD8+ T cells suggested a role for NKG7 in immune synapse formation and/or granule exocytosis, as previously hypothesized (25, 39, 40). To examine this, we used immunofluorescent microscopy to comprehensively assess the subcellular localization of endogenous NKG7 in murine CD8+ T cells at steady state and during synapse formation. While commercial NKG7 antibodies have not previously been used successfully for immunofluorescent microscopy (21, 25), we were able to build on our expertise in staining for granule associated proteins in mouse T cells to optimize immunofluorescent staining of NKG7 (41) (**Supplementary Figure S2**). CD8+ T cells from *Nkg7^-/-^* mice were used as a negative control to confirm the specificity of NKG7 staining (**Supplementary Figure S2**). To determine the subcellular localization of NKG7, we co-stained NKG7 with proteins known to be associated with early endosomes (EEA1), late endosomes (Rab7), late endosomes/lysosomes (LAMP-1) and cytotoxic granules (GzmB) (42). At steady state, NKG7 did not colocalize with EEA1 in early endosomes or with Granzyme B in cytotoxic granules (Pearson’s Coefficient < 0.5) (**Figure 3A**). Rather, the majority of NKG7 colocalized with Rab7 in the late endosomes and, to a lesser extent, with LAMP-1 (Pearson’s Coefficient > 0.5) (**Figure 3A**).

To determine if NKG7 traffics to the immune synapse during target recognition and T cell polarization, we co-cultured activated CD8+ T cells with anti-CD3/28 coated, CellTrace™ Violet-labelled P815 cells and again examined the cellular localization of NKG7 by immunofluorescent microscopy. Translocation of the microtubule organizing center (MTOC) to the interface of T cell/target cell conjugates was used as a marker of synapse formation (43). We found that NKG7 was significantly localized to the polarized MTOC region in CD8+ T cells that had formed a synapse compared to “un-synapsed” T cells where the MTOC was not polarized (**Figure 3B**). Given this marked polarization of NKG7 upon synapse formation, we questioned whether NKG7 was required for polarization of Granzyme B – a key cytotoxic protein that localizes to the T cell/target cell interface following synapse formation for delivery into the target cell. Surprisingly, the absence of NKG7 had no effect on the polarization of Granzyme B following formation of an immune synapse (identified by MTOC polarization) (**Figure 3C**). Together, these data demonstrated that NKG7 polarizes to the immune synapse upon target recognition, but the reduced cytotoxic activity of NKG7 deficient CD8+ T cells does not appear to be due to impaired granule trafficking to the cell interface following synapse formation.

### NKG7 Shortens Immune Synapse Duration and Promotes Efficient Serial Killing

**Figure 4:**
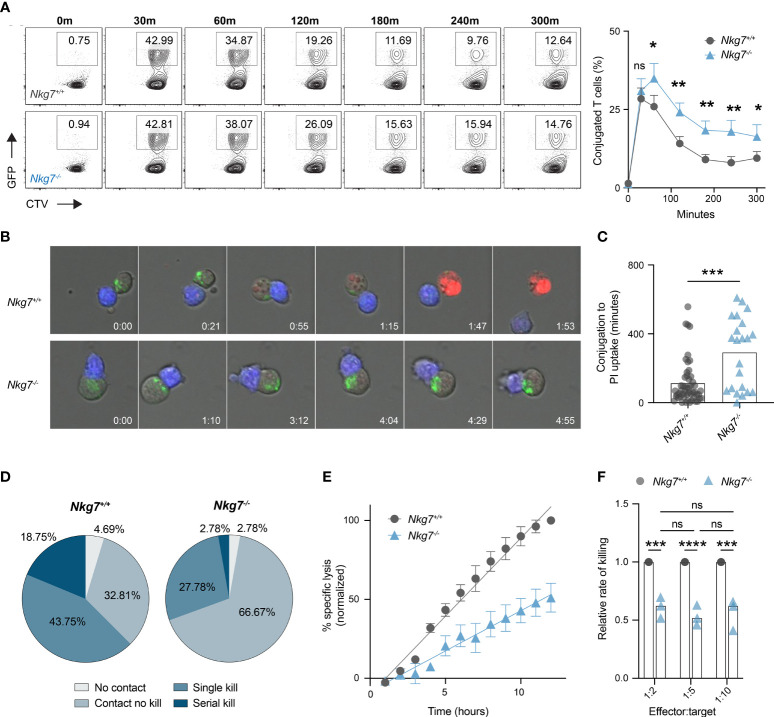
NKG7 shortens immune synapse duration and promotes efficient serial killing. **(A)** Frequency of CD8+ T cells conjugated to P815 target cells at indicated timepoints evaluated by flow cytometry. 72 hour activated *Nkg7^+/+^* or *Nkg7^-/-^* CD8+ T cells were labelled with cell trace violet (CTV) and co-cultured with anti-CD3/28-coated GFP-labelled P815 cells. Time indicates minutes of co-culture. Plots are gated on CTV^+^ cells and frequency of conjugates was calculated as CTV^+^GFP^+^ as a percent of all CTV^+^ cells. Data is quantified in right panel, Wilcoxin matched-pairs signed rank test, n = 3. **(B)** Live cell imaging time-lapse montage of CTV-labelled 72 hour activated *Nkg7^+/+^* or *Nkg7^-/-^* CD8+ T cells (blue) co-cultured with anti-CD3/28 antibody-coated P815 target cells. Lysotracker (green) was added to co-cultures for cell clarity and PI was added to measure target cell death. Time indicates hours of co-culture. **(C)** From **(B)**, time taken between T cell/target conjugation and target cell death as measured by PI uptake, unpaired t test with Welch’s correction, n = 21-55 T cells evaluated, pooled from 2 independent experiments over a 14-hour co-culture. **(D)** Frequency of T cells from **(B)** making no contact with any target (No contact), making contact without killing (Contact no kill), killing a single target only (Single kill) or killing more than one target (Serial kill) during the first 7 hours of co-culture. **(E)** Specific lysis of ^51^Cr-labelled anti-CD3/28-coated P815 tumor cells (targets) co-cultured with 72 hour activated *Nkg7^+/+^* or *Nkg7^-/-^* CD8+ T cells (effectors) at an effector to target ratio of 1:5 for indicated lengths of time, measured by ^51^Cr release. Data was normalized by converting the maximum killing by *Nkg7^+/+^* CD8+ T cells to 100%. **(F)** Relative rate of killing measured from assays set up as in **(E)** at indicated effector to target ratios, with relative rate measured as the slope of the *Nkg7^-/-^* killing curve relative to the *Nkg7^+/+^* killing curve at the corresponding effector to target ratio, 2-way ANOVA Sidak’s multiple comparisons test, n = 3. All error bars show +/- SEM. ns – not significant, * P < 0.05, ** P < 0.01, *** P < 0.001, **** P < 0.0001.

Given *Nkg7^-/-^* CD8+ T cells retain the capacity to form an immune synapse with a tumor target, we next examined whether they exhibited any temporal differences in synapse formation compared to control *Nkg7^+/+^* CD8+ T cells. To do this, we labelled *Nkg7^+/+^* or *Nkg7^-/-^* CD8+ T cells with CellTrace™ Violet, co-cultured them with anti-CD3/28-coated GFP-labelled P815 cells, and analyzed the percent of conjugated cells over time by flow cytometry. We found that both *Nkg7^+/+^* and *Nkg7^-/-^* CD8+ T cells formed contacts with target cells at equal efficiency, with maximum conjugation occurring after only 30 minutes of co-culture in both groups (**Figure 4A**). However, after 30 minutes, a significantly higher frequency of conjugates was observed in *Nkg7^-/-^* T cell-tumor co-cultures, suggesting these cells remain synapsed for longer than *Nkg7^+/+^* T cells (**Figure 4A**). To investigate this further, we applied time-lapse imaging to quantify dynamic differences in synapse formation and duration of *Nkg7^+/+^* and *Nkg7^-/-^* CD8+ T cells in real time. Using PI uptake as a measure of target cell death (2, 5, 38, 44), we quantified synapse duration as the time taken for a target cell to die following conjugate formation. Consistent with our flow cytometry data, we found that *Nkg7^-/-^* CD8+ T cells form a markedly prolonged synapse, taking significantly longer to kill target cells following conjugation (**Figures 4B, C****)**. Proportionally, *Nkg7^-/-^* CD8+ T cells were also less likely to kill a target cell upon contact compared to *Nkg7^+/+^* T cells (66.67% contacts with no kill, compared to 32.81%, respectively), and were less likely to undergo serial killing, with only 2.78% killing more than 1 target, compared to 18.75% of *Nkg7^+/+^* CD8+ T cells (**Figure 4D**).

To further assess if NKG7 enhances the rate of tumor killing, we co-cultured *Nkg7^+/+^* or *Nkg7^-/-^* T cells with anti-CD3/28-coated P815 targets cells at a ratio of 1 T cell (effector) to 5 tumor cells (targets) and measured the percent of tumor targets killed overtime for 12 hours. This low effector:target ratio ensured tumor cells were in excess, allowing T cells to kill at a maximal rate unrestricted over the time course of the assay. We found that the reduced killing capacity of *Nkg7^-/-^* T cells compared to *Nkg7^+/+^* T cells was linearly amplified over time, confirming that *Nkg7^-/-^* T cells kill at a significantly slower rate than *Nkg7^+/+^* T cells (**Figure 4E**). Specifically, we calculated the relative rate of killing of *Nkg7^-/-^* T cells to be 52% of that of control *Nkg7^+/+^* T cells (**Figure 4F**). Notably, as long as tumor cells were kept in excess (at least 2 tumor cells per T cell), the relative rate of killing of *Nkg7^-/-^* T cells was reduced to same level regardless of changes in the number of targets in the culture. This suggested that the reduced rate of killing by NKG7 deficient T cells is not simply due to differences in time to find a target cell (for example, due to reduced migration) but rather impaired intrinsic ability of the T cells to efficiently trigger target cell death upon conjugation.

### Loss of NKG7 Promotes Hypersecretion of Cytokines Following Immune Synapse Formation

**Figure 5:**
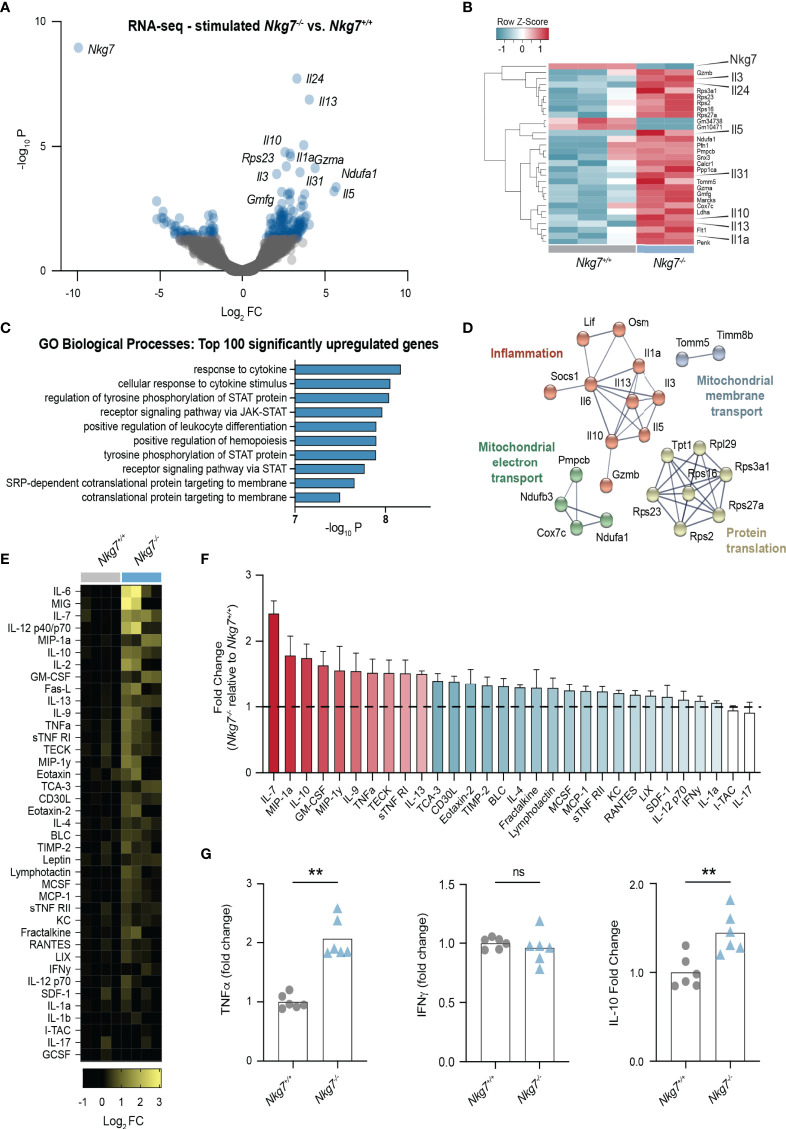
Loss of NKG7 promotes hypersecretion of cytokines following immune synapse formation. **(A–D)** RNA-sequencing of 72 hour activated *Nkg7^-/-^* and *Nkg7^+/+^* CD8+ T cells following co-culture with anti-CD3/28-coated P815 cells for 4 hours. **(A)** Volcano plot of genes up or down regulated in *Nkg7^-/-^* cells compared to *Nkg7^+/+^* cells, with blue indicating genes with a P-value < 0.05, FC – fold change **(B)** Heatmap of top 30 differentially expressed genes. **(C)** Functional Gene Ontology (GO) enrichment analysis of the top 100 differentially upregulated genes. **(D)** STRING network analysis of top 50 differentially upregulated genes, showing connected nodes only. **(E–G)** Cytokines secreted by 72 hour activated *Nkg7^-/-^* or *Nkg7^+/+^* CD8+ T cells co-cultured with anti-CD3/28-coated P815 tumor cells for 4 hours, measured by antibody-pair-based array **(E, F)** or cytokine bead array **(G)** using supernatants from co-cultures. **(E)** Columns represent replicates pooled from 2 independent experiments. **(F)** Red shows cytokines with average fold change >1.5; blue shows cytokines with average fold change >1, <1.5; error bars show +/- SEM. **(G)** Unpaired t test, n = 6; ns – not significant, ** P < 0.001.

Given our observation that *Nkg7^-/-^* CD8+ T cells form a prolonged and inefficient synapse, we next investigated the consequence of this on the transcriptional response of the T cells upon recognition of a tumor target. To this end, we stimulated *Nkg7^+/+^* and *Nkg7^-/-^* CD8+ T cells with anti-CD3/28-coated P815 cells, followed by isolation of the T cells for RNA sequencing. Notably, the most significant differentially downregulated gene in *Nkg7^-/-^* T cells compared to *Nkg7^+/+^* T cells was *Nkg7* itself, thus validating the quality of the data (**Figure 5A, B****)**. Interestingly, among the top upregulated genes in *Nkg7^-/-^* T cells were genes encoding cytokines, including IL-1a, IL-13, IL-5, IL-3 and IL-10 (**Figure 5A, B****)**. Indeed, functional Gene Ontology (GO) enrichment analysis of the top 100 differentially upregulated genes in *Nkg7^-/-^* T cells revealed biological processes involved in cytokine responses and Janus kinase (JAK)-signal transducer and activator of transcription (STAT) pathway signaling (**Figure 5C**). Further network analyses of these genes using STRING identified networks involved in protein translation and inflammatory responses (**Figure 5D**). Together, this suggested that NKG7 deficient T cells transcribe and translate higher levels of inflammatory cytokines following target recognition, which is likely driven through continuous stimulation during a prolonged immune synapse.

To determine if these transcriptional changes were also observed at the protein level, we collected the supernatant from T cell/tumor co-cultures and used cytokine antibody-pair-based and bead arrays to quantify the levels of 30 different chemokines and cytokines. These data confirmed that upon synapse formation, *Nkg7^-/-^* CD8+ T cells secrete higher levels of a number of cytokines including IL-7, IL12, IL-10, IL-2, TNF-alpha and IL-13 (**Figures 5E–G****)**. Surprisingly, we found no significant increase in the production of IFNγ (**Figure 5G**). Together these data suggest that NKG7 deficient CD8+ T cells hypersecrete inflammatory cytokines during a prolonged immune synapse with tumor cell targets.

### Cytokine Hypersecretion in the Absence of NKG7 Compensates for Inefficient Synapse-Mediated Cytotoxicity

**Figure 6:**
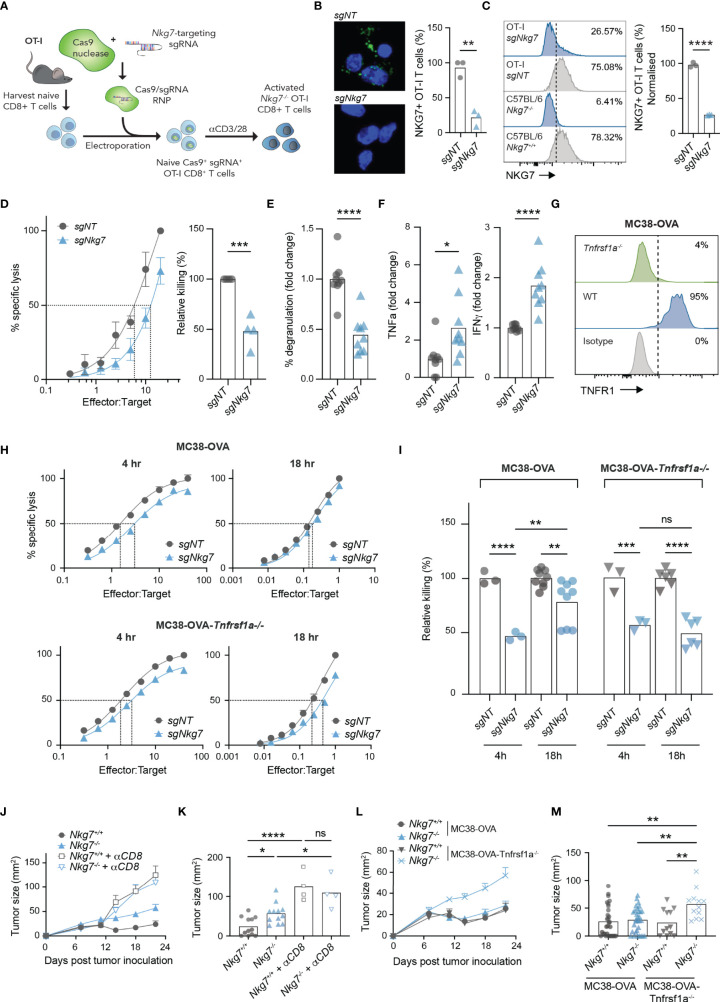
Cytokine hypersecretion in the absence of NKG7 compensates for inefficient synapse-mediated cytotoxicity. **(A)** Schematic of CRISPR/Cas9-mediated knock out of *Nkg7* in CD8+ T cells from OT-I transgenic mice. Naïve CD8+ T cells were isolated and electroporated with Cas9 complexed to sgRNA targeting *Nkg7*. Cells were then activated with anti-CD3/28 antibodies and used for analyses 72 hours post-activation. **(B)** Immunofluorescence microscopy showing NKG7 staining in CD8+ T cells electroporated with a non-targeting sgRNA (*sgNT*) or Nkg7-targeting sgRNA (*sgNkg7*), quantified in right panel, unpaired t-test, n = 3. **(C)** NKG7 protein expression detected by flow cytometry in *sgNT* and *sgNkg7* OT-I T cells, and CD8+ T cells from C57BL/6J *Nkg7^-/-^* and *Nkg7^+/+^* littermate mice, quantified in right panel; percent of NKG7^+^ OT-I T cells normalized for *sgNT* OT-I NKG7 expression equal to 100%, unpaired t-test, n = 3. **(D)** Specific lysis of ^51^Cr-labelled MC38-OVA tumor cells (targets) by *sgNT* or *sgNkg7* OT-I T cells (effectors) in a 4-hour co-culture as measured by chromium release at increasing effector to target ratios. Relative killing (right panel) calculated as the relative efficiency of T cells to achieve 50% specific lysis of target cells, unpaired t test, n = 3. **(E)** Degranulation of *sgNT* or *sgNkg7* OT-I T cells co-cultured with MC38-OVA target cells for 4 hours, measured by OT-I T cell surface exposure of CD107a during the co-culture, detected by flow cytometry, unpaired t test, pooled data from n = 3 independent experiments. **(F)** Cytokines secreted by *sgNT* or *sgNkg7* OT-I T cells co-cultured with MC38-OVA tumor cells for 4 hours, measured by cytokine bead array on supernatants from co-cultures, unpaired t test, pooled data from n = 3 independent experiments. **(G)** TNFR1 protein expression detected by flow cytometry in wild-type (WT) MC38-OVA cells or MC38-OVA cells electroporated with Cas9 complexed to sgRNA targeting *Tnfrsf1a* (gene encoding TNFR1; MC38-OVA-*Tnfrsf1a*^-/-^). **(H)** Specific lysis of ^51^Cr-labelled MC38-OVA or MC38-OVA-*Tnfrsf1a^-/-^* tumor cells (targets) by *sgNT* or *sgNkg7* OT-I T cells (effectors) in 4-hour or 18-hour co-cultures as measured by chromium release at increasing effector to target ratios. **(I)** Relative killing from **(H)**, calculated as the relative efficiency of T cells to achieve 50% specific lysis of target cells, 2-way ANOVA, Tukey’s multiple comparisons test, pooled data from n = 3 independent experiments. **(J)** Tumor growth of MC38-OVA-*Tnfrsf1a^-/-^* cells implanted subcutaneously in *Nkg7*^+/+^ or *Nkg7*^-/-^ littermates with or without CD8 depletion antibodies administered starting the day prior to tumor inoculation, n = 4-12. **(K)** Tumor size from **(J)** on day 22 post tumor inoculation, One-way ANOVA (n = 4-12). **(L, M)** Data pooled from **Figures 1E, F** and panels **(J, K)**. All error bars show +/- SEM. ns – not significant, * P < 0.05, ** P < 0.01, *** P < 0.001, **** P < 0.0001.

To confirm our findings in the setting of an immune synapse formed *via* a TCR-antigen interaction, we next used CRISPR/Cas9 to delete NKG7 in transgenic OT-I CD8+ T cells, by electroporating Cas9/sgRNA complexes into naïve T cells prior to activation (**Figure 6A**) (45). Using this method, deletion of NKG7 protein was achieved with approximately 75% efficiency, as assessed by both immunofluorescent microscopy and flow cytometry (**Figures 6B, C****)**. Consistent with our findings in *Nkg7^+/+^* and *Nkg7^-/-^* littermates, *Nkg7* sgRNA-electroporated OT-I T cells (*sgNkg7*) demonstrated significantly reduced killing of MC38-OVA tumor cells compared to OT-I T cells electroporated with a non-targeting control guide (*sgNT*) (**Figure 6D**). This reduction in killing was also associated with a significant decrease in degranulation (**Figure 6E**). We next used cytokine bead arrays to examine the secretion of TNF and IFNγ by *sgNkg7* and *sgNT* OT-I T cells during synapse with MC38-OVA tumor targets, as these are key cytokines that contribute to the anti-tumor effector activity of CD8+ T cells (10). Interestingly, in this setting, we found an increase in the secretion of both TNF and IFNγ by *sgNkg7* cells compared to *sgNT* controls (**Figure 6F**). This data suggests reduced degranulation and cytotoxicity, as well as cytokine hypersecretion, are consistent phenotypes of NKG7 deficient CD8+ T cells. However, the secreted cytokine profile may vary depending on stimulatory/inhibitory signals received by the T cell, which are likely to vary within the immune synapse across different tumor targets.

Given that MC38-OVA tumors are sensitive to TNF-mediated cell death (4, 5), we hypothesized that hypersecretion of TNF may overcome the reduced immune synapse-dependent cytotoxic activity of NKG7 deficient T cells. Indeed, this would in part explain why the absence of NKG7 does not affect CD8+ T cell mediated control of MC38-OVA tumors *in vivo* (**Figure 1E**). To investigate this, we used CRISPR/Cas9 to delete *Tnfrsf1a*, encoding the TNF binding receptor, TNFR1, in MC38-OVA tumor cells (**Figure 6G**). We then measured the capacity for *sgNT* or *sgNkg7* OT-I T cells to kill MC38-OVA or MC38-OVA-*Tnfrsf1a^-/-^* tumor cells in 4 hours (when tumor cell death is mediated by the perforin-granzyme pathway) or 18 hours (when tumor cell death is mediated by both the perforin-granzyme and TNF pathways) (27). Consistent with our hypothesis, the cytotoxic activity of *sgNkg7* OT-I T cells at 4 hours was significantly reduced against both MC38-OVA and MC38-OVA-*Tnfrs1a^-/-^* tumor cells (**Figures 6H, I****)**. However, by 18 hours, the relative killing rate of *sgNkg7* OT-I T cells was significantly increased against MC38-OVA cells, but remained unchanged against MC38-OVA-*Tnfrsf1a^-/-^* cells lacking TNFR1 expression (**Figures 6H, I****)**. Collectively these data demonstrate that reduced cytotoxic activity *via* the perforin-granzyme pathway in the absence of NKG7 can be compensated by TNF-mediated tumor cell death due to hypersecretion of TNF by NKG7 deficient T cells.

To test this hypothesis *in vivo*, we challenged cohorts of C57BL/6J *Nkg7^+/+^* and *Nkg7^-/-^* littermate mice with MC38-OVA-*Tnfrsf1a^-/-^* tumors and monitored tumor growth, with or without depletion of CD8+ T cells (**Figures 6J, K****)**. As previously observed, there was no difference in tumor growth in either mouse cohorts when CD8+ T cells were depleted (**Figures 6J, K****)**. However, whereas previously we did not observe any significant differences in MC38-OVA tumor growth in *Nkg7^+/+^* and *Nkg7^-/-^* mice (**Figure 1E**), in the absence of tumor cell TNFR1 signaling (as in MC38-OVA-*Tnfrsf1a^-/-^* cells), there was a significant reduction in tumor control in *Nkg7^-/-^* mice compared to *Nkg7^+/+^* littermates (**Figures 6J–M****)**. Together these data demonstrate that hypersecretion of TNF in the absence of NKG7 compensates for inefficient synapse-mediated cytotoxicity to control MC38-OVA tumor growth.

## Discussion

A spate of recent studies have used diverse *in vivo* models to demonstrate a functional role for NKG7 in CD4+ T cells (visceral leishmaniasis), CD8+ T cells (malaria), NK cells (melanoma) (25) and in CD8+ T cell driven anti-tumor immunity (20, 21). Collectively these studies highlight the importance of NKG7 in different immune cell subsets and disease contexts. However, despite a previously described role for NKG7 in CD8+ T cell cytotoxic function (20, 21), we unexpectedly found that CD8+ T cells controlled MC38-OVA tumors at an equivalent rate in wildtype and NKG7-deficient littermate mice, which prompted us to further investigate the functional role of NKG7 in CD8+ T cells. Our analysis uncovered a role for NKG7 in enhancing the efficiency, but not capacity, of CD8+ T cells to form immune synapses with tumor targets and trigger cell death. Indeed, in the absence of NKG7, CD8+ T cells remained capable of killing tumor targets, but at a markedly slower rate. The slow kill rate of NKG7 deficient T cells resulted in a significantly longer T cell-tumor cell synapse, thereby prolonging T cell stimulation, and consequently promoted hypersecretion of inflammatory cytokines. In the setting of a tumor which is sensitive to cytokine-mediated death, cytokine hypersecretion by NKG7 deficient CD8+ T cells compensated for their inefficient synapse-mediated cytotoxicity, leading to a net-zero effect on overall tumor control.

Interestingly, in the 48 hours immediately following activation, we observed that CD8+ T cells briefly downregulated NKG7. This is consistent with a previous report by Ng *et al.* (25) in which NKG7 expression was examined in human CD8+ T cells pre- and 48 hours post-activation. However, by monitoring temporal changes in NKG7 expression over a longer time course, we found that NKG7 is upregulated after 48 hours, and expression continues to increase over time in culture. Notably, our re-analysis of publicly available bulk and single-cell RNA-sequencing datasets also revealed higher levels of NKG7 transcripts in CD8+ T cells with a terminal effector phenotype versus a memory phenotype; the latter of which is associated with an earlier activated state, typified by less differentiated or more stem-like features (36).

Such dynamic expression of NKG7 throughout activation and differentiation may serve two possible functions. Firstly, during T cell activation by antigen-presenting cells (APCs), downregulation of NKG7 may allow T cells to maintain a prolonged synapse with APCs for more robust activation, while also preventing T cell-mediated lysis of the APC. Secondly, NKG7 expression may increase as CD8+ T cells terminally differentiate to favour direct synapse-mediated cytotoxicity, while lessening broader systemic inflammation. Certainly, cytokines play an important role early in the T cell response, where inflammation can serve to both promote target cell death and recruit other immune subsets to the site of the tumor or infection. However, continuous inflammation, such as in the setting of a chronic infection or cancer, can damage normal tissues and ultimately be harmful to the host. It is possible that progressive upregulation of NKG7 in chronically activated CD8+ T cells serves to enhance their direct killing efficiency and minimise the inflammatory damage they may otherwise cause overtime. Consistent with this idea, terminal differentiation or ‘exhaustion’ of CD8+ T cells is associated with a progressive loss in cytokine production (46), which is inversely related to the progressive upregulation we observed with NKG7 expression.

While our study demonstrated that NKG7 deficient CD8+ T cells form a prolonged immune synapse with target cells prior to target cell lysis, the functional role of NKG7 in this process remains unknown. Our findings confirmed previous reports that the absence of NKG7 leads to reduced degranulation (20, 25), which is required for the T cell to deliver toxic cargo (perforin and granzymes) to the target cell to initiate apoptosis (47). It is possible this impaired degranulation simply leads to a prolonged immune synapse due to delayed target cell lysis. However, it is also possible that suboptimal synapse formation itself prevents efficient degranulation (48). Whether NKG7 plays a direct functional role in degranulation, or in fact is critical for efficient formation of a bona fide immune synapse in the first place remains unclear. The latter theory is somewhat supported by a recent report demonstrating that siRNA knockdown of *Nkg7* reduces cell membrane extensions between T cells and tumor cells (20), which could potentially lead to a less stable immune synapse, and inefficient triggering of cytotoxic activity.

Regardless of the order of events, an inevitable result of NKG7 deficiency is delayed T cell detachment from the target. Interestingly, delayed detachment due to impaired target lysis has also been observed in CD8+ T cells lacking perforin (49). In this setting, a prolonged synapse leads to repetitive calcium signaling and, similar to NKG7 deficiency, consequent cytokine hypersecretion. Indeed, there are many parallels between our findings and those observed in perforin deficient CD8+ T cells. However, perforin is a fundamental and indispensable component of synapse-mediated target cell lysis. In contrast, the absence of NKG7 does not completely abrogate T cell cytotoxic activity, but may more subtly fine-tune the synapse to regulate killing activity and inflammation depending on the phenotype of the T cell and stage of disease.

The identification of NKG7 as a regulator of cytotoxic lymphocyte function and inflammatory responses suggests that targeting this molecule may be a therapeutic approach to treat diverse conditions including infection, autoimmune diseases and cancer. However, future studies will need to focus on identifying the proteins NKG7 interacts with within the subcellular compartments of lymphocytes, which may provide new insights into the precise mechanisms behind how NKG7 regulates inflammatory responses in cytotoxic lymphocytes and impacts on disease outcomes.

## Data Availability Statement

The data presented in the study are deposited under BioSample (SAMN28461275 - SAMN28461279) and Bioproject IDs (PRJNA838721).

## Ethics Statement

The animal study was reviewed and approved by Peter MacCallum Cancer Centre Animal Experimentation Ethics Committee (AEEC).

## Author Contributions

Conceptualization: EL, AlB, JO. Methodology: EL, KR, MJ, AlB, AmB, NY and JO. Investigation: EL, KR, CK, JM, IP AmB, NY. Visualization: EL, AmB. Data analysis: EL, AmB, CS, MD. Writing: EL, AmB, JO. Funding Acquisition: JO. All authors read and approved the final manuscript.

## Funding

This work was supported by funding from the National Health and Medical Research Council (NHMRC) of Australia (Grant #1139626) and National Breast Cancer Foundation (Grant #IIRS-18-151) project grants and the Peter MacCallum Cancer Foundation.

## Conflict of Interest

The authors declare that the research was conducted in the absence of any commercial or financial relationships that could be construed as a potential conflict of interest.

## Publisher’s Note

All claims expressed in this article are solely those of the authors and do not necessarily represent those of their affiliated organizations, or those of the publisher, the editors and the reviewers. Any product that may be evaluated in this article, or claim that may be made by its manufacturer, is not guaranteed or endorsed by the publisher.
