# Supplementary material for: NKG7 Enhances CD8+ T Cell Synapse Efficiency to Limit Inflammation
Source: Front Immunol. 2022 Jul 6;13:931630. doi: 10.3389/fimmu.2022.931630 (PMC9299089; doi:10.3389/fimmu.2022.931630)
Supplement: Supplementary file 1 [file DataSheet_1.pdf]

SUPPLEMENTARY FIGURE 1

A Protein expression (antibody derived tags)

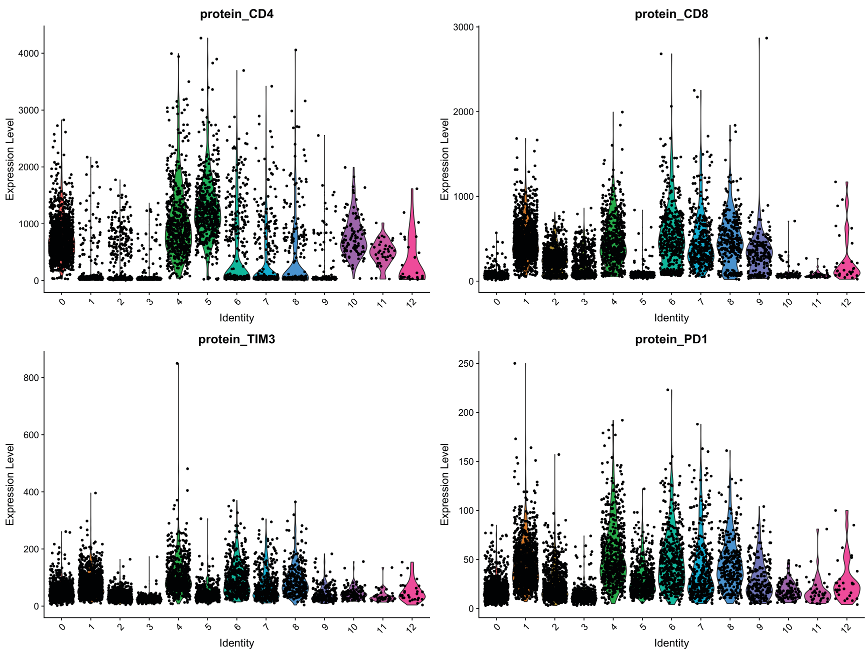

B Gene expression

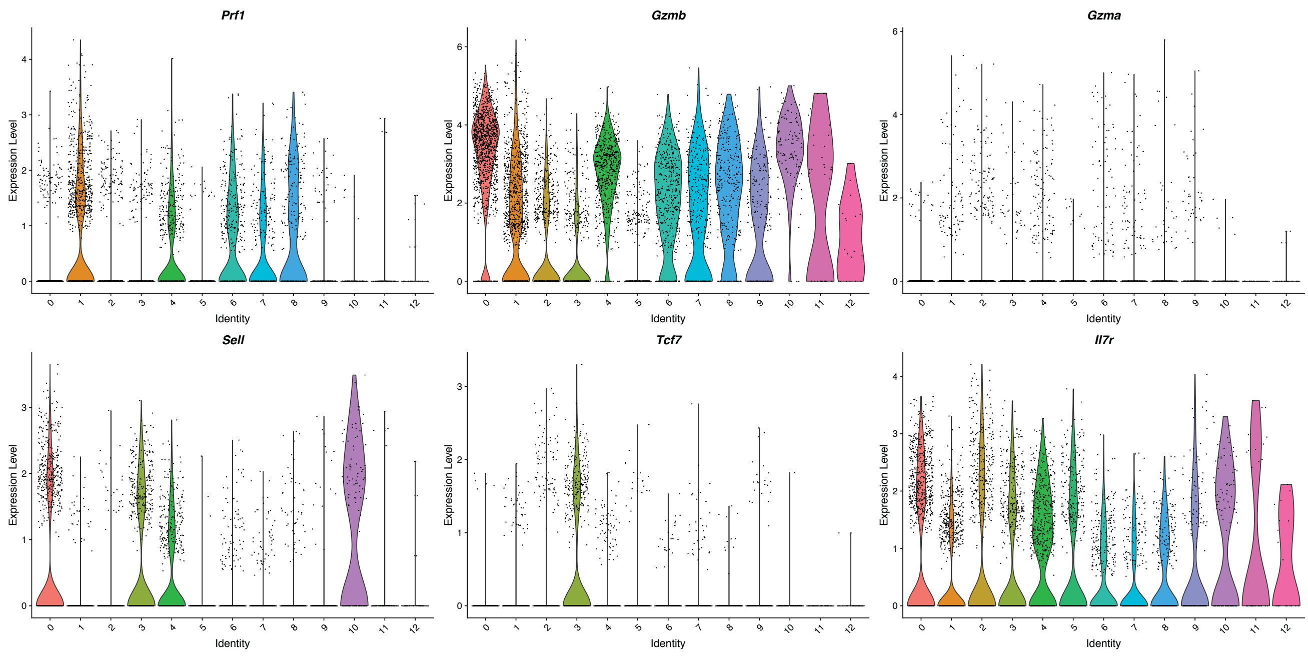

C Goldrath gene signatures

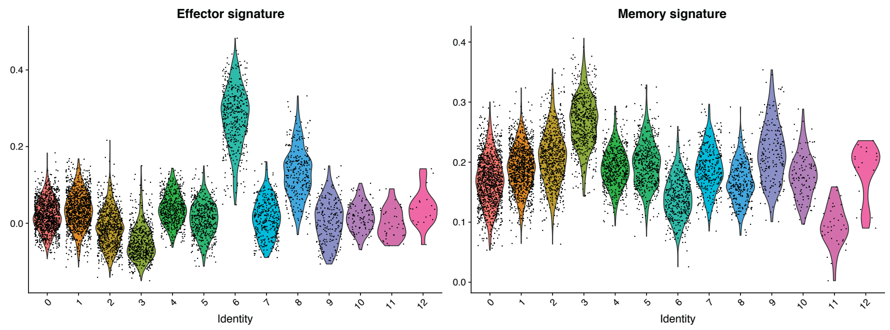

SUPPLEMENTARY FIGURE 1 | Analysis of single-cell CITE-seq dataset used to annotate clusters in Figure 1C, available through the GEO (GSE182664, Lelliott et al, 2021). (A) Protein expression by cluster, as determined by antibody-derived tags. (B) Gene expression by cluster. (C) Goldrath effector and memory signatures takes for Molecular Signatures Database, showing enrichment by cluster.

SUPPLEMENTARY FIGURE 2

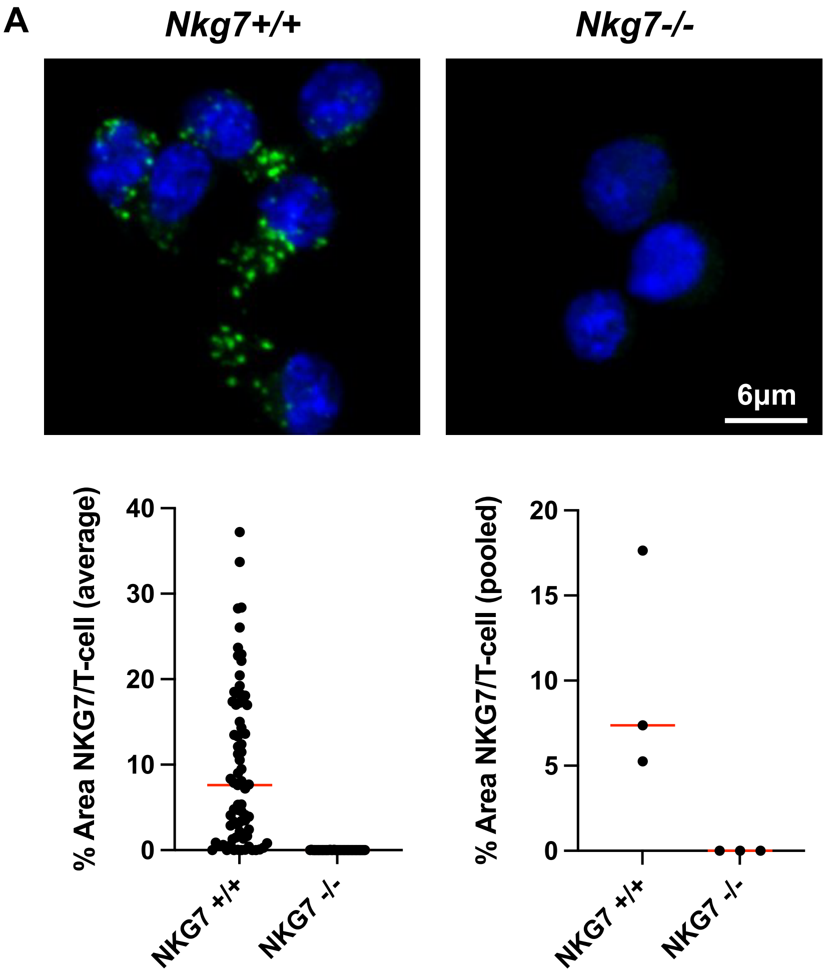

SUPPLEMENTARY FIGURE 2 | NKG7 antibody staining optimization for confocal immunofluorescence microscopy. (Top panel) *Nkg7*$^{+/+}$ and *Nkg7*$^{-/-}$ T-cells were processed identically for immunofluoresence staining (fix/perm; either PFA/escin, PFA/triton or Bouins' solution/escin) and NKG7 protein assessed using the NKG7 antibody (Cell Signalling). Figure is representative images of PFA/escin, identical data was obtained using other fix/perm combinations (data not shown). (Bottom panel) The percentage area of NKG7 staining present in images taken from *Nkg7*$^{+/+}$ and *Nkg7*$^{-/-}$ T cells was determined for each T-cell analysed.
